# Preventive effect of onion juice on selenite-induced experimental cataract

**DOI:** 10.4103/0301-4738.49391

**Published:** 2009

**Authors:** Alireza Javadzadeh, Amir Ghorbanihaghjo, Somayeh Bonyadi, Mohammad Reza Rashidi, Mehran Mesgari, Nadereh Rashtchizadeh, Hassan Argani

**Affiliations:** Department of Ophthalmology, Nikookari Eye Hospital, Tabriz University of Medical Sciences, Tabriz, Iran; 1 The Drug Applied Research Center, Tabriz University of Medical Sciences, Tabriz, Iran

**Keywords:** Antioxidants, experimental cataract, onion

## Abstract

**Purpose:**

To evaluate the effects of onion juice on sodium-selenite induced cataract formation.

**Materials and Methods:**

Thirty-two 10-day-old Wistar-albino rat pups were divided into four equal groups. Group 1 received only subcutaneous saline injection. In Group 2, sodium-selenite (30 nmol/g body weight) was injected subcutaneously. In Group 3, subcutaneous sodium-selenite was injected and one drop 50% diluted fresh juice of crude onion was instilled every 8 h into the right eye for 14 days; the left eye received no treatment. Group 4 rats were similar to those of Group 3, the only difference being that of undiluted fresh juice of crude onion. The development of cataract was assessed. Rat lenses were analyzed for total antioxidant (TA) level, and for activities of glutathione peroxidase (GPX) and superoxide dismutase (SOD).

**Results:**

Both eyes of all rats in Group 1 did not exhibit cataract formation. In Group 2, all rats developed Grade 3 cataract in the lenses of both eyes. The difference in exhibited cataract in the lens of the right eyes in all rats between Group 2 and any eyes of groups 3 or 4 were significant (*P* = 0.001). The mean TA level and mean activities of SOD and GPX in Group 2 rat lenses were significantly lower than the values in lenses of all rats in Group 1 (*P* = 0.001, 0.003, 0.001), and in the lenses of the right eyes of rats in Groups 3 and 4 (*P* = 0.001, 0.020, 0.001).

**Conclusion:**

Instillation of onion juice into the rat eyes can effectively prevent selenite-induced cataract formation. This effect was associated with increased TA level, SOD and GPX activities in the lens.

Cataract is a major health problem and the major cause of blindness throughout the world.[1] Currently, the only available treatment for the disease is the surgical extraction of the cataractous lens followed by replacement with a synthetic implant. Although such a surgical replacement of the natural lens with an artificial lens is significantly effective in restoring vision to most patients, it is not free of complications. Attempts to prevent cataract formation, or at least significantly retard the onset of the disease would be of great value.[2]

There is a large body of evidence indicating the role of oxidative stress and radical oxygen species in the mechanism of cataract development and opacification process.[3,4] According to this hypothesis, free radicals are capable of perturbing the homeostasis of lens leading to loss of transparency.[4] It has been suggested that hydrogen peroxide (H_2_O_2_) can act as a major oxidant in human senile cataract.[2] This oxidizing agent is present in normal aqueous humor at concentrations of approximately 20–30 μM and could be raised up to 660 μM in patients with cataract.[5] Cui *et al.*, have shown that *in vitro*, hydrogen peroxide at these higher concentrations can cause lens opacification and damage similar to that found in human cataract.[6] Therefore, it can be postulated that the prevention of reactive oxygen production or scavenging of free radicals would be an effective strategy to prevent or delay cataract formation or progression. This strategy, in particular through the use of nutritional antioxidants, has received much attention from researchers.[7–11] There are some studies indicating that the disturbance in the oxidative state of the lens can be corrected by giving various antioxidants.[12] Devamanoharan *et al.*, have demonstrated the prevention of selenite-induced cataract development by ascorbic acid.[13] The effectiveness of physiological antioxidants such as pyruvate and nutritional antioxidants such as ascorbate, vitamin E, and carotenoids in delaying the development of experimental cataract has been reported by some investigators.[13–16] According to Jacques *et al.*,[17] individuals with high plasma levels of vitamin C, vitamin E, and carotenoids appear to have reduced risk of cataract.

Flavonoids are a group of substances that are ubiquitously distributed in various foods and beverages of plant origin.[18] These naturally occurring compounds have strong antioxidant properties[19] and the potential of dietary flavonoids in the prevention of cataract has been shown by some studies.[3,20] Orhan *et al.*, have demonstrated that propolis (a flavonoid-rich natural waxy mixture produced by honey bees) and quercetin, a major dietary flavonoid, are capable of preventing selenite-induced cataract formation to the extent of 70% and 40%, respectively.[12] In an *in vitro* study, quercetin was found to be an effective inhibitor of H_2_O_2_-induced experimental cataract in rat model.[2] The protective effect of tea, a major source of dietary quercetin and other flavonoids, has been reported by Rechner *et al*.[21] The ability of isoflavone genistein in delaying the progression of cataracts induced by dietary galactose has been reported by Huang *et al*.[22]

The onion, (*Allium cepa*), is a staple food with a high content of flavonoids.[23] The aim of the study was to assess whether topical instillation of fresh onion juice could prevent cataract formation in selenite-induced experimental cataractogenesis in rat model, and the status of total antioxidant (TA) level, and activities of the enzymes glutathione peroxidase (GPX) and superoxide dismutase (SOD), as a marker of oxidative stress in explanted rat lenses.

## Materials and Methods

Thirty-two Wistar-albino rat pups, housed with their mother, were divided into four groups (three experimental and one control), each consisting of eight pups. Experiments were conducted in accordance with the ARVO Statement for Use of Animals in Ophthalmic and Vision Research and Guiding Principles in the Care and Use of Animals. The fresh juice of crude onion was prepared each time before instillation by peeling ripe, red, fresh, dried (median weight 25–50 g) onions and grinding them for about 10–15 min in a mechanical blender. The resulting mash was then filtered (Nalgene sterile bottle filters), and the filtrate centrifuged (3000 rpm, 4C°) until obtaining a relatively clear supernatant liquid. The juice was next diluted in water in order to prepare 50% diluted onion juice. Group 1 received only subcutaneous saline injection and was the control group. In Group 2, sodium-selenite (30nmol/g body weight, Sigma Chem. Co., St Louis, USA) was injected subcutaneously on postpartum Day 10. In Group 3, subcutaneous sodium-selenite (30 nmol/g body weight) was injected on postpartum Day 10 and one drop 50% diluted fresh juice of crude onion (red onion) was instilled every 8 h into the right eye, starting one day before sodium-selenite injection (on postpartum Day 9) and was continued for 14 days (till postpartum Day 23). The procedures performed on Group 3 rats were also performed on Group 4 rats, the difference being that undiluted fresh juice of onion was used in place of the diluted juice. Interpalpebral fissures of all rats were opened by a knife under surgical microscope magnification (Topcon OMS60, Japan) on postpartum Day 8. The development of cataract was assessed weekly for two weeks after initiation of treatment, and its density was graded and photographed with a photo-slit-lamp (Topcon Digital Camera Unit DC-1, Japan). One person (A J), masked to the treatment groups, performed all examinations. The left eyes of rats in Groups 2 to 4 were used as an internal control.

At the final examination, the pupils were dilated with tropicamide 0.5% and staging of the selenite-induced cataract was performed by slit-lamp biomicroscopy on a scale of 4 to 0. Grade 4 was a mature dense opacity involving the entire lens; Grade 3 was a strong nuclear cataract with an opacity in the perinuclear area; Grade 2 was a nuclear cataract; Grade 1 was a subcapsular opacity; and Grade 0 was a normal clear lens.[24] All rats were killed 14 days after injection, and their lenses were removed intracapsularly via an incision 2 mm posterior to the limbus under surgical microscope magnification. The lenses thus removed were analyzed for TA level and activities of GPX and SOD. Lens tissues were minced on a glass and homogenized by a glass–glass homogenizator in cold physiological saline on ice. Then the tissues were diluted with a ninefold volume of phosphate buffer (pH = 7.4).[25,26] GPX and SOD activities were spectrophotometrically measured with RANSEL and RANSOD test kits, respectively (obtained from Randox Laboratories Ltd). TA was determined in the lens with the RANDOX kit.

All statistical analyses were carried out using SPSS statistical software (SPSS for windows, Version12, Chicago, IL, USA). Results were expressed as mean ± S.D. All data were analyzed statistically using the Mann-Whitney or the Wilcoxon signed ranks test as appropriate. A *P* value less than 0.05 was regarded as significant.

## Results

**Figure 1:**
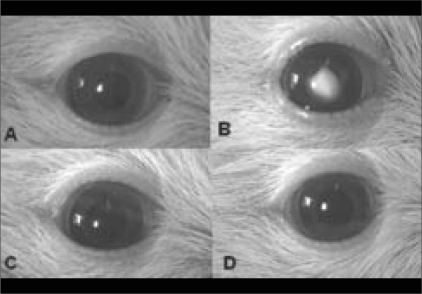
Topcon photo-slit-lamp images of rats’ eyes 14 days after injection (a) Clear lens in control group (b) Grade 3 cataract in sodium-selenite group (c) and (d) Clear lens in sodium-selenite + 50% diluted onion juice and sodium-selenite + onion juice groups

Lenses in both eyes of all control rats (Group 1) remained clear [Fig. 1a]. In Group 2, all rats developed Grade 3 cataract in lenses of both eyes [Fig. 1b]. In Group 3, the lens of the right eye of all rats was clear, except for the lenses of two rats which exhibited cataract of Grades 1 and 2 [Fig. 1c]; In Group 4, the lens of the right eye of all rats was clear, except for the lenses of two rats which exhibited Grade 2 cataracts [Fig. 1d]. The lenses of the left eyes of all rats exhibited cataract (Grade 3) in Groups 3 and 4. No toxic effects to the cornea or conjunctiva of the eye were observed following the application of the juice. The difference in exhibited cataract in the lens of the right eyes in all rats between Group 2 and any groups of 3 or 4 were significant (*P* = 0.001).

**Figure 2:**
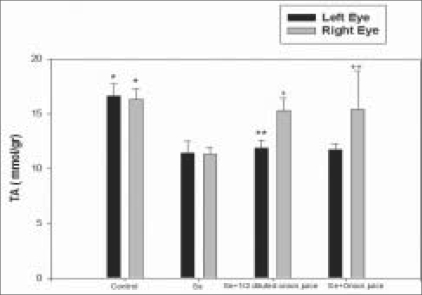
Mean TA level in both eyes of rats 14 days after injection in four experimental groups (* = As compared with Se group *P* = 0.001, ** = As compared with Se group *P* = 0.248, + = As compared with Se group *P* = 0.001, ++ = As compared with Se group *P* = 0.001, TA = Total antioxidant, Se = Selenite)

**Figure 3:**
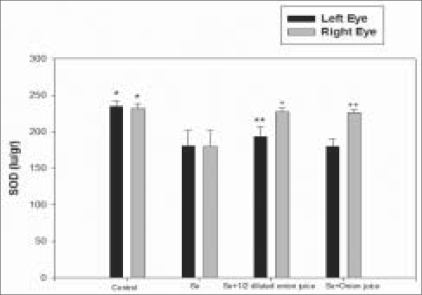
Mean SOD activity in both eyes of rats 14 days after injection in four experimental groups (* = As compared with Se group *P* = 0.003, ** = As compared with Se group *P* = 0.097, + = As compared with Se group *P* = 0.018, ++ = As compared with Se group *P* = 0.020, SOD = Superoxide dismutase, Se = Selenite)

**Figure 4:**
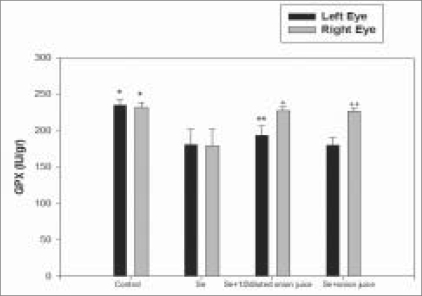
Mean GPX activity in both eyes of rats 14 days after injection in four experimental groups (* = As compared with Se group *P* = 0.001, ** = As compared with Se group *P* = 0.086, + = As compared with Se group *P* = 0.001, ++ = As compared with Se group *P* = 0.001, GPX = Glutathione peroxidase, Se = Selenite)

The mean TA level was 11.3 ± 0.7 mmol/g in the lenses of the right eyes of Group 2 rats; 15.3 ± 1.1 mmol/g in the lenses of the right eyes of Group 3 rats and 15.4 ± 1.0 mmol/g in the lenses of the right eyes of Group 4 rats [Fig. 2]. The mean activity of SOD in the lenses of the right eyes was 27.5 ± 5.0 IU/g (international unit per gram) in Group 2 rats, 34.2 ± 5.6 IU/g in Group 3 rats and 34.6 ± 6.0 IU/g in Group 4 rats [Fig. 3]. The mean activity of GPX in right eye lenses was 179.5 ± 22.2 IU/g in Group 2 rats, 227.3 ± 5.1 IU/g in Group 3 rats and 226.6 ± 3.5 IU/g in Group 4 rats [Fig. 4]. The differences in mean TA level and mean activities of SOD and GPX between Group 2 and Groups 3 (*P* = 0.001, 0.018, 0.001) or 4 (*P* = 0.001, 0.020, 0.001) were significant. No significant difference was observed between the lenses of the right and left eyes, in terms of clinical and biochemical findings in either the control (Group 1) rats or the group that received only sodium-selenite (Group 2 rats) [Figs. 2–4].

## Discussion

Currently, surgical removal of opaque lens is the only treatment for human cataract; however, because of the enormous cost of this operation, many attempts have been made to find other preventive or treatment regimes to delay formation of senile cataract. Human cataract is a multifactorial disease associated with several risk factors. It has been suggested that oxidative stress is involved in cataractogenesis as a common underlying mechanism, and augmentation of the antioxidant defenses of the lens has been shown to prevent or delay experimental cataract.[4] Selenite-induced oxidative stress (20–30 nmol/kg body weight of selenite) causes nuclear opacity through the calpain proteolysis of lens proteins. It is a strong sulfhydryl oxidant and is considered as a model for those cataracts caused by oxidative stress.[27] Similar to human senile cataract, this type of cataract is accompanied by a decrease in activities of the antioxidant enzymes such as SOD and GPX.[28,29] Accordingly, it has been postulated that antioxidant agents can prevent cataract development.[30–32] An increase in free radical species in the aqueous humor and significant reduction of GSH content in cataractous lens have been demonstrated by some authors.[33–35] GSH protects the lens from oxidative damage and also maintains the functions and transparency of the lens by protecting the protein sulfhydryl groups from oxidation.[20] A study demonstrated that the prevalence of all types of cataract was 40% and 80% lower in persons with moderate and high antioxidant index scores compared with persons with low scores.[36] Many studies have shown that flavonoids, as a group of natural compounds with antioxidant properties, can prevent oxidative damage and experimental cataract progression.[20,37–39] Onion is a flavonoid-rich staple food and the major flavonoids have been identified as quercetin, quercetin-4′-glucoside and quercetin-3,4′-diglucoside.[40,41] In a survey of 62 edible tropical plants performed by Miean *et al.*, onion ranked highest in quercetin content.[42]

In the present study, it has been shown that the instillation of fresh juice of crude onion into the rat eyes could prevent selenite-induced cataract formation by 75%. This effect was associated with higher mean TA level and mean activities of GSH and GPX in the lenses of rats receiving fresh juice of crude onion and subcutaneous injection of sodium-selenite compared with those rats which received only sodium-selenite injection [Figs. 2, 4]. It could be postulated that onion juice, as a flavonoid-rich source, can provide an additional support to the antioxidant agents, leading to the elevation of TA levels and GPX and SOD activities in the rat lens, in spite of exposure to sodium-selenite. Orhan *et al.*, have also showed that a nutritional antioxidant such as vitamin C can prevent selenite-induced cataract formation by 41% on the 15^th^ day of the experiment in Wistar rats.[12]

No significant difference was observed between the lens status of right and left eyes in terms of clinical and biochemical findings in the control rats and the group of rats that received only sodium-selenite. However, the status of the lens in the right eyes of those rats that received sodium-selenite and fresh onion juice was significantly better, in clinical and biochemical parameters, compared with the left eyes. These results indicate that onion juice can reach into the lens through the cornea and aqueous humor. The absorption was limited only to the eyes that received the juice.

In summary, the present study has demonstrated that instillation of onion juice into rat eyes can effectively prevent selenite-induced cataract formation. This effect is associated with increase in the activity of SOD and GPX, and in the level of TA in the lens. The results provide information that can be used for designing further studies to investigate the effect of onion consumption in the prevention of cataract formation. This study has some limitations. First, it is impossible for a human to use onion juice directly. Second, because of differences in sources of different kinds of onions we cannot generalize the results of our study to all kinds of onions. Finally, because of the small number of sample size in this study, we need to carry out other studies with more samples.
